# Centering female agency while investigating contraceptive use: a case study in Agincourt, South Africa

**DOI:** 10.1186/s12939-019-0965-7

**Published:** 2019-05-02

**Authors:** Cara Margherio

**Affiliations:** Center for Evaluation & Research for STEM Equity (CERSE), University of Washington, Box 353340, Seattle, WA 98195 USA

**Keywords:** Contraception, Abortion, Family planning, Premarital fertility, Unplanned pregnancy

## Abstract

**Background:**

Rural areas of South Africa face persistently high teenage and premarital childbearing rates, reflecting a lack of or inconsistent use of modern contraception. In attempting to understand this behavior, much of the literature has denied agency to young women, portraying them solely as victims of their environments. This study moved beyond these approaches to understanding adolescent contraceptive use, to reframe the investigation to focus on the tension around exercising agency within specific structural constraints.

**Methods:**

Findings are based on a qualitative study in Agincourt, South Africa. Data were collected through six focus group discussions with 63 women aged 18–44. A grounded theory approach utilizing emergent coding was performed focusing on the decision-making processes around family planning. The focus group participants discussed attitudes and norms around: early first births, contraceptive use, unplanned pregnancy, abortion, and HIV testing. When possible, differences that emerged around these topics according to the age groups (18–24, 25–34, and 35–44) and/or nationality of village (South African or Mozambican) are highlighted.

**Results:**

Participants of all focus groups agreed that early first birth were common and undesirable. Younger participants described pregnancy prevention as a key rationale for contraceptive usage, while older participants were more inclined to cite HIV prevention. Women of all focus groups discussed the importance of women taking the initiative with family planning. Participants expressed a range of opinions about the acceptability of abortion, and all focus groups discussed concerns about the safety of abortion. Finally, all of the focus group participants stressed the importance of HIV testing, both to protect themselves and to protect their families.

**Conclusion:**

This study found many locations of agency for young women in rural South Africa. The decision-making surrounding contraceptive use consists of a series of decision junctures at which women must assign values to certain factors and then select their behavior on the basis of those values. Young women weigh the costs and benefits of contraception and of pregnancy, while also taking into account the chances of actually becoming pregnant along with the costs and benefits of abortion. Furthermore, the women explicitly viewed contraception as within their own realm of decision-making and action (as opposed to within the realm of their male partners).

## Centering female agency while investigating premarital fertility: A case study in Agincourt, South Africa

South Africa has the lowest fertility rates of any country in sub-Saharan Africa and is well into its fertility transition. However, the country, particularly in rural areas, faces persistently high teenage and premarital childbearing rates [1, 2]. In attempting to understand this behavior, much of the literature has denied agency to the young women of rural South Africa, removing decision-making processes from the young women themselves and portraying them solely as victims of their environments. For example, previous research studies have identified several reasons why adolescents in rural South Africa fail to use modern forms of contraception, including: stigmatization of adolescents accessing family planning programs, a lack of communication from families and teachers, perceived lack of risk, and gendered power dynamics in which young women feel they cannot refuse sexual advances or insist on condom usage [3–6]. While identifying valid constraints on modern contraceptive use, these reasons fail to take into account young women’s agency.

While there has been much prior research on the structural constraints influencing use of contraception, there is a surprising lack of research examining how young women exert agency within these structures. Agency may take the form of accommodation and/or resistance to existing rules, norms, and structures; to be an agent involves exerting some level of control over the social environment in which one is enmeshed [7, 8]. Sewell [7] argues that while the level of control varies across individuals, all actors “exercise some measure of agency in the conduct of their daily lives” (p.20). Structure does not determine behavior, but rather influences it; structure and agency are dynamically interconnected [7, 9].

Schatz [10] argued that much of the AIDS literature presents women as without any control of their sexual environments and in need of assistance to improve their autonomy. Schatz’s [10] study utilized qualitative data from Malawi to identify sites where women enact agency by creating and deploying HIV prevention strategies that are more suitable for within their contextual situation. Similarly, the analysis of this study moves beyond traditional approaches to understanding low modern contraceptive use among adolescents by investigating the tension around exercising agency within specific structural constraints. In doing so, this study reframes the conversation around adolescent contraceptive use from one based on women’s vulnerability to one based on women’s ability to exercise agency within structural constraints.

### Family planning in South Africa

Apartheid-era policies have had a lasting impact on contraceptive use and family planning services. During apartheid, the majority of the Black population was forced onto rural homeland areas, separated by ethnicity, that were lacking in employment and agricultural opportunities [6, 11]. Over time the apartheid government became increasingly concerned with the growing size of the Black population and subsequently established the National Family Planning Programme in 1974, to increase contraceptive access for all women, although it was targeted specifically at Black South African women [12, 13]. Injectable forms of contraception were heavily promoted for Black women as they require little user involvement and few follow-up services [12, 14], and they remain the primary form of birth control used by Black women today [15]. Injectable contraceptives are particularly popular among younger users and users in rural areas in South Africa; while users consider this method to be effective and convenient, previous research has also found that recommendations by clinic health personnel were a major reason for their use [16].

The 1996 Choice on Termination of Pregnancy Act legalized abortion on request up to 12 weeks gestation, and up to 20 weeks gestation in cases of incest, rape, socio-economic hardship, and for reasons related to the health of the pregnant women and the fetus. Abortion past 20 weeks may only be performed to save the life of the mother. Surgical termination of pregnancy (i.e. mechanical uterine evacuation) may only be performed by a doctor or trained midwife in facilities designated for that purpose by the Provincial Department of Health, while medical (i.e. non-surgical) termination of pregnancy may be legally performed anywhere. Medical termination of pregnancy in South Africa is commonly performed using misoprostol tablets; this medication is relatively inexpensive and widely stocked in pharmacies for the treatment of peptic ulcers, meaning these tablets are accessible to the general public without medical supervision [17]. Even when women are not harmed by self-induced abortion, this process often results in incomplete or failed abortion attempts, which may then contribute to women seeking later-term abortions, which is associated with an increased risk of complication [18, 17].

High levels of premarital fertility signal high rates of unprotected sexual activity among young people, a high-risk behavior for the spread of HIV/AIDS [19]. Previous research indicates many young South Africans engage in sexual risk-taking behaviors, including early sexual debut, unprotected sexual activity, low levels of condom and contraceptive use, and concurrent partners [20]. South Africa in particular has been heavily impacted by HIV/AIDS; from 1990 to 2005, the HIV prevalence rates soared from less than 1% to around 19% [21]. In the Agincourt area of South Africa, HIV prevalence rates are as high as 45.3% among men and 46.1% among women aged 35–39 [22].

### Setting

This study is nested within the Agincourt Health and Demographic Surveillance System (AHDSS) site in the Ehlanzeni District in Mpumalanga Province, northeastern South Africa. The AHDSS has collected demographic information on a population of approximately 100,000 individuals in 28 villages since 1992. One third of this population is former Mozambican refugees, who self-settled in the area after fleeing civil war. During the apartheid period the Agincourt area was part of the Gazankulu homeland and was thus particularly economically disadvantaged [23, 24]. Due to a dry climate and poor soil, agriculture is poorly developed in the area; the local economy is based on remittances from migrant workers, pensions, and tourism [25]. The majority of economically active adult men are migrant workers; over 60% of men aged 30–39 are migrant workers and live at least part of the year elsewhere [25]. Since the end of apartheid female labor migration has increased; by 2011 more than 30% of women 30–39 were engaged in temporary migration [25]. Similar to the rest of South Africa, many of the lower income families in the AHDSS rely on child support grants for assistance with childcare and school fees. Started in 1998, child support grants are administered by the South African Social Security Agency and consist of income payments to South African citizens who are the primary caregiver of a child and are in need of financial assistance.

Agincourt is nearing the end of the fertility transition. The total fertility rate (TFR), representing the average number of children born per woman, dropped from 3.7 in 1993 to a low of 2.3 in 2002 and has hovered between 2.5 and 2.4 since then [26]. In spite of these declines, persistently high premarital fertility rates have been well documented in Agincourt [3, 5, 24, 27, 28]. Garenne, Tollman, Kahn, Collins, and Ngwenya [3] found a bimodal fertility pattern suggesting many women give birth at a relatively early age prior to marriage and then wait several years before bearing a second child.

Five years after the legalization of abortion, Garenne, Tollman, Kahn, Collins, & Ngwenya [3] found that abortion in Agincourt was usually self-administered as a home remedy, as abortion services were not readily available in rural areas. In a series of focus group discussions in both Agincourt and Soweto, Kaufman, de Wet, and Stadler [1] found that teenage mothers did not discuss abortion in terms of religious beliefs, as abortion was viewed more as an act of desperation when facing an unintended pregnancy as opposed to being viewed primarily as a moral issue. However, they found that religious beliefs did play a role in framing abortion in focus group discussions with both young men and the parents of teenage mothers.

This study uses focus group discussions to investigate women’s attitudes around contraception, family planning, abortion, and HIV testing in Agincourt, South Africa. Of particular interest is furthering the understanding of the persistence of premarital and teenage childbearing throughout the area. The analysis is structured through a focus on the tension between women’s agency and structural constraints surrounding family planning and reproductive health.

## Methods

Focus group discussions were conducted in May 2013. Focus group discussions are appropriate methods for this study, as the data they produce includes insight into complex behavior, motivation, and decision-making processes [29]. Further, these discussions serve to identify issues related to family planning that are salient to many women within Agincourt. Approval was given by both the Human Subject Division, University of Washington, Seattle, United States and the Committee for the Protection of Human Subjects, University of Witwatersrand, Johannesburg, South Africa. Two local fieldworkers were hired to assist with recruitment, facilitation, transcription, and translation, as the focus groups were held in the local language, Shangaan. Focus group participants gave written authorization to the informed consent, which was read to them in full by one of the fieldworkers at the start of each focus group discussion. The focus groups were structured around the topics of: education, work, family planning, unplanned pregnancy, and HIV/AIDS.

Sixty-three (63) total women participated in six separate focus groups. Participants were recruited from six villages: Khaya Lami, Kildare C, Newington B, Newington C, Rholane, and Somerset. Subjects were recruited while they were collecting water from community water taps and walking throughout the village streets. Interested women were eligible to partake in a focus group discussion as long as they were within the age range of the study (ages 18–44). If an interested but ineligible (due to age) individual was recruited, they would be asked to recommend someone who was eligible; thus, we occasionally visited people’s homes or workplaces during the recruitment process. The fieldworkers kept notes throughout recruitment and at the end of each recruitment day would write out a through description of their interactions and procedures. On the day prior to a focus group discussion, all the participants recruited for that discussion would be called via telephone to confirm their attendance.

As noted above, one third of the population within Agincourt is of Mozambican origin. The proportion of residents that identify as Mozambican varies across the villages within the AHDSS site from a high of 95.49% to a low of 2.65%. As shown in Table 1, residents in three of the villages included within this study predominately identify as Mozambican (according to AHDSS census data), while residents in the other three villages included within this study predominately identify as South African. Although we did not recruit based on an individuals’ nationality, this recruitment process allows for a comparison of social norms surrounding family planning between predominately South African and Mozambican villages within Agincourt. The participants were further divided into separate focus groups based on age (18–24, 25–34, and 35–44) for a total of six focus groups ranging from eight to thirteen participants (i.e., 18–24 year-old women from a predominately South African village, 18–24 year-old women from a predominately Mozambican village, etc.). All six focus groups followed the same semi-structured format, regardless of age group or nationality of village. Throughout the findings section, quotations are labeled by an individual identifier (e.g., W1), age group, and nationality of the village (SA for South African or MZ for Mozambican).

**Table 1:** Mid-Year 2012 demographics for six villages within Agincourt, South Africa

Village	Total Population	Percent Mozambican
Predominately Mozambican		
Kildare C	1184	93.50
Rholane	2339	94.74
Somerset B	1182	77.92
Predominately South African		
Khaya Lami	2182	8.85
Newington B	3533	8.94
Newington C	1945	2.78

All of the focus groups were held within centrally located daycare centers or schools within the villages; they were held in the late afternoons after the children had left for the day, school had ended, and individuals had finished work. As participants arrived the fieldworkers and I would greet them; they would talk amongst themselves until we began. To start each focus group one of the fieldworkers would read over the consent forms and answer any questions. After signatures on the consent forms had been collected, we would begin audio-recording the discussion. One of the fieldworkers would moderate the focus group discussion, in Shangaan, while the other fieldworker and I took observational notes. All of the focus group discussions were quite lively with the participants eager to participate and voice their opinions and experiences. The participants would often disagree with each other and ask the other participants to explain their points of view, while the facilitator guided the topics of discussion. The focus group discussions last from 90 to 120 min. After at least two of the focus groups, the participants asked if we would return to conduct further discussions; many of the participants thanked us, telling us how much they enjoyed the experiences, sharing their opinions, and learning new information. At the end of each focus group we distributed cold beverages, rolls of bread, and fresh fruit; this is the standard incentive procedure used by the AHDSS during focus group discussions. We also distributed two pamphlets to each participant, one that addressed HIV testing and treatment options and one that addressed options when facing unplanned pregnancy.

The audio-recordings of each focus group discussion were transcribed and translated by the fieldworkers. Any translation issues that emerged would be discussed and occasionally AHDSS staff would be consulted to ensure accurate translation. The transcriptions were then typed and loaded into Atlas.ti where they were coded in an iterative process using emergent coding. Emergent codes included: contraception and irregular menstruation, abortion and infertility, and HIV and caregivers. Field notes were consulted to ensure accurate interpretations of the participant interactions. Analytic memos were developed on core themes and used to synthesize findings into a summary analysis.

## Results

The focus group participants discussed attitudes and norms around: early first births, contraceptive use, unplanned pregnancy, and HIV testing. When possible, differences that emerged around these topics according to the age groups (18–24, 25–34, and 35–44) and/or nationality of village are highlighted. A summary of these comparisons is shown in Table 2.

**Table 2:** Emergent themes by focus group

Discussion	Mozambican			South African		
	18–24	25–34	35–44	18–24	25–34	35–44
Early first births						
Interrupt schooling and work	x	x		x	x	
Difficulty balancing school and motherhood	x			x	x	
Burden on grandparents			x			x
Financial burden	x	x	x	x	x	x
Ideal to wait until can support a child	x	x	x	x	x	x
Ideal to wait until have a partner	x	x	x	x	x	x
Contraceptive use						
Start prior to dating and after menstruation	x			x	x	
Start at sexual debut		x	x		x	x
Pregnancy prevention as primary rationale	x	x		x		
HIV prevention as primary rationale			x		x	x
Women must take initiative in use	x	x	x	x	x	x
Traditional methods of contraception	x	x	x	x	x	x
Modern methods of contraception	x	x	x	x	x	x
Switching methods due to weight gain or feeling ill	x			x		
Switching methods due to irregular menstruation	x	x	x	x	x	x
Switching methods due to decreased sex drive			x			x
Concerns about infertility	x	x	x	x	x	x
Unplanned pregnancy						
Seek abortion from pride, fear of parents, school	x			x		
Seek abortion for socioeconomic reasons	x	x	x	x	x	x
Seek abortion due to infidelity			x		x	x
Abortion as dangerous	x	x	x	x	x	x
Disagreement around abortion	x	x	x	x	x	x
Abortion and future infertility	x	x	x	x	x	x
Disapproval of abortion from community	x	x	x	x	x	x
Disapproval of abortion from nurses	x	x	x	x	x	x
HIV testing						
Likelihood of partner being unfaithful	x	x	x	x	x	x
Need to get tested if sexually active	x	x	x	x	x	x
At risk of HIV because family caregiver			x			x
Need to get tested to protect family	x			x		
Women need to know their status	x	x	x	x	x	x

### Early first births

While the consensus across all of the groups was that early first births are common, they were also viewed negatively. It was noted that an early first birth interferes with school and could “destroy” a young woman’s future. Younger women were more likely than the older women to focus on early births interrupting schooling and future work prospects, often reflecting on their own experiences. For example, one participant stated, “Like myself, I got a child while I was 14 years and I’m not willing that my child could be the same as me. I didn’t complete my school, and it has disturbed me a lot.” [W5 25–34 MZ] However, the women discussed the difficulty of balancing school and young motherhood, indicating that young mothers did frequently return to school after childbirth. One woman described the challenges of young mothers returning to school by stating:

I don’t think it is good to have a child when you are still young because you will be still at school and you will have to leave your child at home and your child will not be treated in a proper way. By the time you pass grade 12 you will want to go to tertiary, while your child is still young, and you have to leave them at home. The child will never learn to love you as their mother because you didn’t have enough time to bond with your child and the child needs their mother’s love. [W2 18-24 MZ]

As noted in the above quotation, it was expected that the children of young mothers would be left at home with their grandparents as caretakers while the mothers returned to school and/or work. The two focus groups comprised of women aged 35–44 years framed their discussion of teenage mothers around the suffering the situation brings to grandparents. One participant stated that, “As a parent it will be difficult to you because you have to carry a burden of being a grandmother and look after your grandchild.” [W4 35–44 SA] In a different focus group a participant explained that the caretaking would also present a financial burden, stating, “[I] t is difficult because if she is going to school she must stay … If the parent was cleaning somewhere, she must leave it to stay with her grandchild and you find that there is nothing to eat at home. It is difficult. [W2 35–44 MZ] Although the older women were concerned about the burden teenage motherhood created for grandmothers, they still felt it was necessary for the young women to finish schooling after an early first birth.

Early first births were viewed as financial burdens, despite child support grants. The women from Mozambican villages discussed the need for proper identity documents proving citizenship or permanent resident status in order to obtain the child support grant. One woman explained:

There are no benefits in getting a child while still young, even if there are child support grants. And it is not enough nowadays because we are using pampers, not napkins, and they are expensive. Sometimes it might happen that you don’t even have an identity document, so how are you going to register your child support grant? Because of that you will suffer. [W6 18-24 MZ]

There was agreement across all of the focus groups that it is ideal to wait to have a first birth until the woman is mature and in a position to support the child, whether through her own work or through her husband’s employment. As one participant stated that, “I will plan to have a first child when I’m working because I will be able to maintain him or her.” [W1 18–24 SA] In the same focus group, another participant noted that, **“**I can plan to have a child if I’m married and my husband is working.” [W4 18–24 SA] The majority of participants stressed finding a partner before becoming pregnant; one participant stated, “If I can find a man who is working I can plan to have a child.” [W11 18–24 SA] The importance was placed on having a partner and not on having a formalized marriage. As one participant explained:

Even if it is not white wedding, as long as the girl has told her parents that she found someone who wants to marry her and the marriage is introduced to both families. I don’t think it will be a problem if the girl can fall pregnant because she will know who is responsible for her pregnancy. [W7 35-44 SA]

Thus, rather than placing the focus on a formal marriage (whether for religious, moral, or legal reasons), the focus was placed on knowing who would be responsible for the child.

Given the lack of male employment opportunities, it was not surprising to hear many of the women focus on the need for themselves to be employed before their first birth. For example, one participant stated, “Even if I’m not married, when I’m working I can [have a child].” [W6 35–44 MZ] Given that all focus groups stressed the importance of delaying childbearing until women are partnered and working, exploring these norms did not help explain the persistent trend of early first births. To analyze this issue further, norms and behaviors surrounding contraceptive use were also investigated.

### Contraceptive use

Differences between the groups around when to first seek contraception revealed differences in their opinions of the primary rationale for using contraception. In the focus groups of 18–24 year-olds, the participants agreed that women should seek contraception both prior to dating and once they begin menstruation; they were likely to cite pregnancy prevention as the primary rationale for using contraception. One of the younger participants explained how her reasoning was based on her own experience, stating, “16 years is too late, because I fell pregnant when I was 14 years. It’s better to start using contraceptives when you have started seeing your periods, because if you have started to menstruate you will start dating.” [W1 18–24 SA] Older women were more likely to suggest that females should seek contraception at sexual debut, citing HIV prevention as an important rationale. For example, one woman noted:

It is good for a young woman to start using family planning if she has already started to have sex, because nowadays there are diseases. When it comes to protection or family planning she needs to know what kind of family planning she can use in order to prevent diseases. [W2 35-44 SA]

As shown in this quote, the older participants tended to link family planning and HIV prevention.

Women in all of the focus groups discussed the importance of females taking initiative with family planning; contraception was viewed as a locus of female agency, albeit with numerous institutional constraints. For example, one participant emphasized:

If you have started dating you should go for family planning, because the boy is not going to tell you to go for family planning. As a girl you have to think by yourself if you don’t want to fall pregnant. [W6 25-34 SA]

Refusing sexual activity was seen as one option, as stated by a younger participant, “If he doesn’t want to use a condom you mustn’t have sex with him.” [W1 18–24 SA] When engaging in sexual activity, the women stressed the need to remain aware of their male partner’s actions, as described in the following conversation [18–24 SA]:

W9: If you are busy having sex you need to concentrate on what you are doing so that you can feel if the condom has burst or not. As a girl you need to differentiate between sex with condom and sex without condom. You must not only enjoy sex without feeling the difference.

W11: If you are having sex you don’t have to enjoy it only you must also concentrate so that you can feel it if the condom has burst.

W9: When you have sex you must be active.

W1: If you have sex you need to be active, because you have decided to use a condom for your own safety you must watch what your husband or boyfriend is doing.

In this dialogue, the women discussed the need to exert agency by being proactive, within the context of a lack of trust of their male partners.

When discussing where contraceptive methods were obtained, women were just as likely to mention visiting clinics as home methods and traditional healers. Traditional methods of contraception were discussed in-depth; younger women in particular were aware of riskier home methods, such as drinking manganese.^1^ Many of the women discussed how their mothers and grandmothers taught them these methods. For example, one participant stated, “I have been told by my grandmother that you can use ash. You need to use it early in the morning after sex before you sweep the yard.” [W11 25–34 SA] Traditional healers were also seen as being able to prevent pregnancy, as explained by this participant, “There’s also a traditional contraceptive when you are menstruating, they take the [menstrual] pads that you are using and put a muthi [herbal medicine] on it, and dig a hole where they keep it.” [W10 18–24 MZ] The women went on to explain that this is believed to prevent conception as long as the menstrual pad remains buried.

In discussing traditional methods of contraception, the participants emphasized female agency, even to the extent of hiding methods from their male partners. For example, one woman described a traditional method as follows:

Other people are using warm water to prevent pregnancy. Every time after sex you just drink a cup of warm water. You must make sure that there is water in your room because immediately after you finish having sex you must drink it. It might happen that your partner asks you why you always drink water when you finish having sex. You need to tell him that you are thirsty. [W6 18-24 MZ]

Regardless of which type of contraceptive method the women were discussing, the emphasis was placed on remaining in control: the females, and not the males, were seen as the source of contraceptive knowledge and decision-making.

In discussing reasons women may switch between contraceptive methods or stop using them altogether, the focus groups of 18–24 year-old women discussed weight gain, feeling ill, and concerns about infertility. Adverse reactions to injectable contraception were noted multiple times, such as by this participant, “Like myself, when I use injectables I will always have lower abdominal pains so the best contraceptive for me is to use a condom.” [W1 18–24 SA] Another participant noted, “Let’s take the person is using a Depo-Provera, an injectable that lasts for three months, and it makes her to have a continuous menstruation. She is going to change to another type.” [W5 18–24 SA] The young women from predominately Mozambican villages were equally knowledgeable about modern methods of contraception and adverse reactions to injectables. For example, one participant explained. “There are two types of injectables: Nur-isterate, it lasts for two months, and Depo-Provera, it last for three months. Other people, if they use Nur-isterate they have continuous menstruation.” [W5 18–24 MZ].

All of the focus groups discussed irregular menstruation as a common reason to switch or stop using contraceptive methods, particularly in regard to injectable contraception as noted above.

The women in the two focus groups comprised of women aged 35–44 were the only ones to discuss a decreased sex drive as a deterrent from using contraception. For example, one participant stated:

I had an opinion on why someone would change the type of contraception she is using. There are some types of contraception that kill your feelings. Even if your husband touches you, you are feeling nothing. So, if you can come across this situation, you need to change the type of contraception you are using to another that can make you feel when your husband touches you. [W6 35-44 SA]

As seen in this quotation, the women discussed feeling agency over their sexual relationships in addition to agency over family planning.

Participants also discussed fears of contraceptive-caused infertility as a barrier to contraceptive use. One participant warned, “Family planning is not good when you are still young because you might end up not conceiving.” [W11 18–24 SA] Fears of infertility surrounded both modern and traditional methods of contraception. For example, one participant explained:

If you use traditional contraceptives, the one of taking a pad you are using when you are menstruating and put some muthi [herbal medicine] on it, digging a hole, and putting it in there. If the traditional healer who did it for you dies and they didn’t show you where they put, it you won’t be able to have children. [W2 18-24 MZ]

Furthermore, traditional methods of contraception were also discussed in regard to their unreliability; one woman stated, “If you use traditional contraceptives you are at a higher risk because you don’t know how your reproductive system works and you don’t even know how strong your husband’s sperm are.” [W9 18–24 SA] In both of the focus groups composed of women aged 18–24, a few of the participants covered their mouths with their hands and giggled while the other women explained traditional methods of contraception, indicating their disbelief. This reaction did not occur among any of the participants in the other focus groups.

### Unplanned pregnancy and abortion

While, as noted above, participants were quite knowledgeable regarding multiple contraceptive options, participants in all of the focus groups stated that unplanned pregnancies are common. One participant stated that, “99% of women don’t plan [first births] and 1% do plan.” [W3 18–24 MZ] It was further noted that women may plan for later births but that the first birth was generally unplanned. One potential cause was the apparent lack of parental communication regarding initiation of contraception. One woman attributed her own early first birth to this, stating:

I fell pregnant at an early age, but I was using some contraceptives. Sometimes I was scared to go to the clinic because I was doing it without permission from my mother. I was also scared to tell her that I have started seeing my periods… Because of being scared to tell my mother, I had a child who is now 8 years old because I fell pregnant at the age of 15. [W6 18-24 MZ]

This participant described exerting agency though seeking modern contraceptive use at the clinic. She also outlined how a lack of parental communication and fear of her mother’s disapproval created a challenging context within which to exert her agency 100 % of the time.

Abortion as an option when facing unplanned pregnancy was also noted in all of the focus groups. The reasons a woman would seek to obtain an abortion differed significantly by age group. The 18–24 year-old participants cited pride, fear of telling one’s parents, and the desire to finish school as the reasons a woman would seek an abortion. The older women were more likely to note socioeconomic challenges and infidelity as the reasons a woman would seek an abortion. Older women and women from predominately South Africa villages were significantly more likely to say that abortion was justifiable under certain circumstances. For example, one woman stated, “if you committed adultery while you are married and scared that your husband will find out there is no option other than terminating the pregnancy.” [W7 35–44 SA] Women from predominately Mozambican villages were more likely to view abortion as a religious sin.

All of the participants, and older women in particular, viewed abortion as dangerous. Some of the participants saw abortion as dangerous for religious reasons. For example, one woman stated, “It is dangerous to terminate the pregnancy as it might happen that God will punish you. Instead of the baby dying, you will die. I will never allow my daughter to have an abortion.” [W5 35–44 SA] Other women explained that in spite of religious reasons, abortion was sometimes still justifiable. One woman explained:

She can think it is a sin, but the situation forced her to terminate … It might happen that I find I am pregnant meanwhile I am ill. It could be impossible to stay with the pregnancy. Maybe she is a child, still young, and she gets pregnant and she is not having any help. It is possible she can terminate the pregnancy because it is difficult to stay with that pregnancy because it cannot take her anywhere. [W2 35-44 MZ]

In a few of the focus groups, heated discussion erupted on the subject of abortion, as the participants held a wide-range of opinions on the matter. Overall, these tensions centered on viewing abortion as a religious sin versus viewing abortion as unavoidable in some circumstances.

As with contraception, fears of future infertility also arose while discussing abortion. One of the younger women stated, “But there is this thing of going to the clinic and terminating the pregnancy. Later when you get married, your husband will demand a child and you will fail to give him children.” [W3 18–24 SA] The fear of abortion damaging future fertility was also discussed by the older participants; one woman said:

When I fall pregnant I will terminate the pregnancy, but there will be a time where I will find a man of my dreams who will marry me only to find that I can no longer have children. It happens in life and there a lot of issues like that. I will keep on crying and say ‘if’, and that ‘if’ will be useless. [W4 35-44 SA]

Participants in all of the focus groups agreed that abortion was a topic of gossip and that the community would be angry with a woman for obtaining an abortion. Women feared negative repercussions from the community, as one woman explained:

The community and the Induna [community leader] will become angry with you, sometimes they can beat you or expel you from the community as they will think that you will teach young girls who will want to get married and give birth in the future. [W1 35-44 SA]

Participants also viewed this sort of condemnation coming from the nurses at the local clinic. One woman explained the difficulty in obtaining an abortion from a clinic as follows:

The nurse who is responsible for doing TOP [termination of pregnancy], he is no longer willing to do it, but it’s his profession. He has no choice; really, he is no longer interested in even giving you the pill that they use to clean the womb. He will say they are finished meanwhile they [the pregnancy] are still there. He will even ask you why you didn’t go for family planning in order to prevent pregnancy. I once came across a clinic sister who was giving health talks and said that you can do TOP as you wish, but you need to know that you are damaging yourself. She continued saying that the machine used to clean the womb damages something in your womb because when somebody is busy doing TOP you will hear her crying as if she is giving birth. [W1 35-44 SA]

Thus, the attitudes and behaviors of the nurses created barriers for women seeking abortion. The focus group participants expressed frustration at the judgements from the nurses; these judgements in turn served to undermine trust between the women and their healthcare providers.

### HIV testing

While the participants of this study were wary of visiting clinics for abortions, they did view the clinics favorably as sources of HIV testing and treatment. The participants in all of the focus groups stressed the need to get regular HIV tests due to the likelihood of unfaithful sexual partners. For example, one participant stated, “It is important to get tested for HIV, because while you might know that you are a person who doesn’t sleep around meanwhile your partner sleeps around.” [W9 25–34 SA] Suspecting, or even expecting, your partner to be unfaithful occurred regardless of marital status; another participant noted, “[If] you have a husband or you are married, maybe you don’t trust him, maybe he is cheating on you, even if you are healthy, it makes you to go and test in order to know your status.” [W11 25–34 MZ] Additionally, participants in all of the focus groups noted the necessity of being tested after participating in unprotected sexual intercourse. One woman explained:

[She should get tested when] she started dating and when she has started to have sex, even if she is 12, 15, 19 or 20 years as long as she knows that she has started to have sex and she is not using a condom. [W1 35-44 SA]

The participants all stressed that a person’s age did not matter regarding getting tested for HIV; sexual activity was discussed as the primary reason to seek HIV testing.

Participants were also quick to stress that women need to be tested even if they are not sexually active, because they are vulnerable in their role serving as caregivers for family members who may be infected. For example, one of the women explained:

And even if you are not doing mats [engaging in sexual activity] there are others that you find are sick or injured, you haven’t got time to go and get gloves. You find yourself holding her meanwhile you are also injured, you can also get infected by that way, by that virus. [W7 35-44 MZ]

The women in both focus groups of participants aged 18–24 felt it was important to get tested in order to protect their families. For example, one participant explained:

It is good to do an HIV test at an early stage because you will be able to protect those who are close to you. If you tested in time it will help your family members because when you become sick, even if you are not bed-ridden, when you are unable to bathe yourself they can bathe you. But if you didn’t disclose your status to your family it might happen that the person who bathes you has sores and you are also having sores. When they bathe you, she might get infected because the fluids that come out of your sores might come to her own sores. If you have tested at an early stage, you are able to confide in your family. They will know what to do when you become sick. In that way you have protected your family, your husband, and also your children. [W8 18-24 MZ]

In all of the focus groups, the need to know one’s status was emphasized repeatedly. It was seen as the responsibility of the women to know their status, so that they could act accordingly and exert control over the spread of HIV.

## Discussion

Participants discussed women’s agency in regards to contraception and family planning through: taking responsibility for contraceptive use, exhibiting knowledge about a variety of methods (both traditional and modern), providing biological explanations for switching/ terminating methods (e.g. irregular menstruation, aches and pains), and discussing how young women need to figure it out as their parents will not talk to them about it. The women also discussed several family planning strategies exhibiting the use of agency: refusing sexual activity if the male partner refuses to use a condom, remaining aware during sexual activity in case the condom breaks or the man removes the condom, and hiding contraception from one’s partner.

This study found many locations of agency for young women in rural South Africa. The participants spoke of decision-making processes in which women are acting in what they believe to be in their best interests, although often in the presence of faulty or incomplete information (particularly in regard to ineffective traditional methods of birth control such as drinking a glass of warm water after sexual activity). The decision-making surrounding contraceptive use of sexually active young women consists of a series of decision junctures at which women must assign values to certain factors and then select their behavior on the basis of those values. Young women must weigh the costs and benefits of contraception and of pregnancy, while also taking into account the chances of actually becoming pregnant along with the costs and benefits of abortion. In calculating the costs and benefits of contraception, the women in this study discussed: structural factors such as barriers to obtaining contraception, the social costs of acknowledging sexual activity (especially high costs for younger women), and biological factors such as the impact of contraception on menstrual cycles. Contrary to the findings of Preston-Whyte, Zondi, Mavundla, and Gumede [30] the participants in this study do view their fertility as within their control. Although the women in the study discussed the costs of pregnancy, particularly as an obstacle to completing schooling, they also spoke to the struggle to consistently use contraception. The participants, of all ages, were firm and unanimous that there were no benefits to early childbearing.

Participants in all of the focus groups were quite knowledgeable about both modern and traditional contraceptive methods. Traditional methods, which several of the women acknowledged are less reliable, are often easier to access than modern methods which require regular clinic visits. Thus, the risk of traditional methods of birth control may at times outweigh the benefit of efficacy of modern contraception. Furthermore, the women viewed contraception as within their own realm of decision-making and action (as opposed to within the realm of their male partners). Similarly, although the participants did not feel they could control if their partner was unfaithful, they found agency in the ability to seek HIV testing; getting tested and knowing your status are important forms of agency within these constraints.

The findings presented here support the bimodal fertility pattern found by Garenne, Tollman, Kahn, Collins, & Ngwenya [3], reflecting a lack of modern contraception prior to first birth, a low prevalence of abortion, and high contraceptive prevalence following first birth. This study found that stigmatization of adolescent sexuality, fear of parental disapproval, lack of communication from parents regarding contraception, and disapproval from nurses all created structural constraints to the use of modern forms of contraception. These findings are in line with prior research showing that when adolescents do seek family planning services, they often find that they are unwelcome at the health clinic [1, 23, 31]. Neglect, rudeness, and physical assault by nurses have been widely reported, especially within sexual and reproductive health services [23, 32]. It is possible that for those young women who had an early first birth, the process introduced them into the reproductive healthcare system, thus reducing the costs of future contraceptive use. It is therefore important to incorporate these women into the reproductive healthcare system at younger ages in order to decrease the rate of adolescent childbearing. It would also be quite valuable to examine the education that the nurses receive, revising this curriculum to discourage stigmatizing adolescent sexual activity in efforts to increase adolescents’ access to contraception.

This study has some limitations. Results from this rural, former homeland are not representative of the general population. It is likely that women’s experiences with family planning in urban areas and in rural areas that were not former homelands will be different than what was presented here. This research is based on data collected only from women participants. Further research is needed to incorporate the experiences and opinions of men, including both their understandings of women’s agency and their own agency around early first births, family planning, and HIV testing. Reframing the conversation beyond acknowledging the barriers women face in accessing contraception to investigating the complex decision-making that influences contraceptive choice allows for researchers to broaden their understanding of family planning and how agency operates for these young women within the institutional confines of their context.

## Footnotes

1 Informational pamphlets concerning the safety of traditional methods of contraception were prepared based on these research findings and distributed to the women within Agincourt by the AHDSS office.
